# Peer Review of “Pentavalent Vaccine: How Safe Is It Among Infants Accessing Immunization in Nigerian Health Facilities (Preprint)”

**DOI:** 10.2196/66894

**Published:** 2024-10-17

**Authors:** Daniela Saderi, Musa Ali, Paul Hassan Ilegbusi, Toba Olatoye, Shah S

**Affiliations:** 1 PREreview, Portland, OR, United States, 1 123456789; 2 Hawassa University, Hawassa, Ethiopia; 3 Ondo State College of Health Technology, Akure, Nigeria; 4 University of Ilorin, Ilorin, Nigeria; 5 IGDORE, Karachi, Pakistan

**Keywords:** Nigeria, vaccines, pediatrics, infants, safety, immunization

*This is a peer-review report submitted for the preprint “Pentavalent Vaccine: How Safe Is It Among Infants Accessing Immunization in Nigerian Health Facilities.”*

This review is the result of a virtual collaborative live review discussion organized and hosted by PREreview and JMIR Publications on August 22, 2024. The discussion was joined by 19 people: 2 facilitators, 1 member of the JMIR Publications team, 3 authors, and 13 live review participants, including 5 who agreed to be named: Randa Salah Gomaa Mahmoud, Femi Qudus Arogundade, Anand Gourishankar, Dr Nour Shaballout, and Queen Saikia. The authors of this review have dedicated additional asynchronous time over the course of 2 weeks to help compose this final report using the notes from the live review. We thank all participants who contributed to the discussion and made it possible for us to provide feedback on this preprint.

## Summary

### Research Question

The study [1] aims to assess the safety of administering the pentavalent vaccine to infants in Nigeria by evaluating the incidence and severity of adverse events following immunization (AEFIs). The main objective is to provide local evidence via surveillance on the vaccine’s safety among infants administered in Nigerian health facilities, providing evidence that the benefits of vaccination outweigh any potential risks.

### Research Approach

A prospective observational approach was employed to actively monitor AEFIs, with clear inclusion and exclusion criteria for recruitment to ensure the reliability of the data. The research conducted used a stratified random sampling method across 16 health facilities in Abuja, Nigeria, ensuring geographic representation from primary, secondary, and tertiary levels. A total of 423 infants received three doses of the pentavalent vaccine at 6, 10, and 14 weeks. Mothers and caregivers were trained on how to identify AEFIs. The mothers and caregivers were also provided with diaries and thermometers to monitor and record AEFIs after and between each vaccine dose. They were encouraged to take their infants to the doctor in case of serious AEFIs, with a follow-up conducted via telephone to ensure accurate data collection.

### Research Findings

The findings identified various AEFIs, commonly including pain, fever, swelling at the injection site, vomiting, refusal to feed, excessive crying, coughing, rash, diarrhea, restlessness, and severe local reactions. However, study findings revealed that AEFIs were generally mild, and their incidence decreased in frequency with subsequent doses, with no significant differences based on gender. No severe adverse events were reported, and the vaccine was well accepted by mothers and caregivers. Despite a follow-up rate of 55.5%—meaning that AEFI data for 235 infants was received—the study documented reasons for the loss to follow-up. The reasons for the loss to follow-up provide valuable insights for future avenues of research, with regard to policy implications and immunization practices. The findings support the continued use of the pentavalent vaccine in routine immunization programs, contributing to improved public health within the Nigerian health care context.

### Research Implications

The study concluded that the pentavalent vaccine is safe for infants, supporting its continued use in Nigeria’s routine immunization schedule. The results are particularly important for ensuring continued public trust in vaccination programs by utilizing the existing surveillance system. This has broader implications for regions where vaccine safety is a major concern. The study’s real-world applicability to the Nigerian context enhances the generalizability of its findings and underscores the importance of continuous short-term safety monitoring. While the study highlights the gap for long-term safety monitoring, its focus on Nigeria provides valuable data on the vaccine’s safety in a West African context. By considering the interactions of environmental, health care, and genetic factors, we can better understand the influence of both AEFIs and outcomes for infants receiving the pentavalent vaccine in addition to other routine immunizations.

### Main Strengths

The study’s recruitment method effectively synchronized the age of infants at 6 weeks at the start of the study for a number of participants in a context where the exact date of birth and age are often unreliable, which is impressive. Furthermore, the research focuses on identifying major risks associated with pentavalent vaccines using a “prospective active observation” method, which is both simple and comprehensive as compared to more resource-intensive and complex methodologies used in studies with similar objectives. While this study was conducted as a coordinated multicenter study, the observed decrease in AEFIs with subsequent vaccine doses suggests that the infants may adapt to the vaccine, offering important insights for health care providers when counseling parents about AEFIs and routine vaccinations.

### Main Weaknesses

However, a significant weakness lies in the study design, which may not be the most suitable for assessing the active monitoring component follow-up of the research objective. A case-control study design might have been more appropriate to further ascertain if educating mothers and caregivers on nonserious and serious AEFIs, providing diaries and thermometers for monitoring, and encouraging them to seek help in the case of serious AEFIs resulted in better reporting of AEFIs than the mothers and caregivers without the counseling, educating, and resources. Additionally, the study falls short in analyzing the long-term impact of AEFIs, as the data found primarily mild self-limiting reactions. No long-term effects were reported or serious AEFIs that necessitated hospital visits either. This limitation could hinder a complete understanding of the vaccine’s safety profile. Another noted weakness is the pie chart used for Figure 1, which provides absolute numbers without percentages or details of which AEFIs occurred, the dose, or where (facilities).

Below, we present a list of major and minor concerns and feedback as they were highlighted during the live review discussion and further elaborated on by the reviewers who compiled this review.

## List of Major Concerns and Feedback

- The control groups here may be the mothers/caregivers whose infants qualified to receive routine immunization around the same time but did not meet the inclusion criteria of the study and so did not receive the same counseling, education, and resources with regard to serious and nonserious AEFIs. The reviewers believe that more detailed information on the comparison between groups in this study is needed.

- It might be useful to discuss any correlations observed as well as explain how confounding variables were addressed by authors to better understand the methodology. Providing a detailed timeline of all the concurrent vaccines administered during the study period and explaining measures taken to differentiate adverse effects from different vaccines will only make the study stronger and clearer that it is studying a component of surveillance known as active monitoring with specific activities.
- It is stated that SPSS was the software used for data analysis, but there are no other details about the specific statistical methods or procedures employed. More information about the statistical analyses performed in the study would be very helpful. For example, the authors report that “...the sex of the infants did not exhibit statistically significant differences in the types or occurrence of reported AEFIs during our study,” but the reviewers could not spot the statistical test results (values) for the comparison anywhere in the manuscript. If those are reported somewhere and we missed them, we suggest they are made more clear in the text and the figures. Moreover, a list of which statistical tests were used for each analysis and why should be provided in the Methods section.

- Although the study was powered for a sample size of 423, there is no mention of how/if the study results, which included only 235 infants, are adequate for analysis at this level of significance, especially as the minimum according to their calculation is 385. In the results, it is mentioned that “This [235 infants] sample size was deemed sufficient for drawing conclusions regarding the safety of pentavalent vaccines, allowing for comparisons with findings from other studies.” What is the rationale behind this statement?
- There appears to be no information/introduction on whether the methods and tools for counseling, monitoring, evaluating, and reporting the AEFIs have been previously used and adapted to this context (in which case there should be a reference) or if they were newly implemented (in which case it would be helpful to see details of piloting and report internal validation results of tools, especially given the translation in different languages).
- The reviewers thought that having measurement ranges for fever and clear definitions for pain and other variables explained to the mothers and caregivers would be very helpful to better understand how the data was categorized. For example, was “irritation” separated from “pain,” and how was that explained to the caregivers?
- The site of vaccine administration or the nature of the vaccine that was given (eg, live attenuated vaccine) was not specified. This information is especially important as “pain” and “swelling” were the most frequently reported local AEFIs.

## List of Minor Concerns and Feedback

- The introduction would be strengthened by providing an overview of the routine childhood immunization schedule in Nigeria.
- Making the data, protocol, and code used for the analysis available would greatly increase the likelihood that other researchers reproduce the experiments in their own settings. For example, it would be very helpful to have more detailed information about what resources were used to train mothers/caregivers to identify and categorize serious and nonserious AEFIs, and how they were counseled on monitoring guidelines and encouraged to bring the infants to health facilities in case of serious AEFIs. Perhaps the authors may consider including the format used to collect data, study variables, and timetables as an appendix. Furthermore, information on what was covered in the phone calls would be very useful. For example, the reviewers wondered if the scope of the phone calls was just to check monitoring and documentation, or if the mothers/caregivers were assessed for their knowledge and understanding about serious and nonserious AEFIs and methods of data collection. More information on the follow-up questions would be useful for others wishing to replicate a similar study. Finally, it appears that the sociodemographic data about the caregivers were collected. Can those data be made available?

- The reviewers wondered if mothers/caregivers were properly informed about the fact that their consent or refusal to be part of the study would not compromise or enhance the quality of the care received. Providing the informed consent sheet might be useful to showcase this.
- The reviewers wondered if apart from monitoring the mothers’/caregivers’ reports of AEFIs, doctors also visited and clinically assessed the status of the infants during the subsequent visits (at both 10 and 14 weeks). If so, is that data/information available? It would also be useful to know whether the infants were examined directly before receiving each vaccine dose as part of the study protocol.
- The reviewers wondered if mothers/caregivers were asked to report on the use of antipyretic medications, and if not, it would be useful to discuss this as a potential limitation.
- The preprint does not clearly define the time range covered by this study, limiting the ability to contextualize the findings and assess their timeliness and relevance.
- Please provide the total number (N) of male and female participants in Table 2.
- The authors say that the data received was classified according to urban and rural in Figure 1, but this information was not found anywhere in the manuscript.
- Results around the prevalence data would be best presented in the Results section rather than in the Discussion section.
- The reviewers wondered if different brands/types of pentavalent vaccines were used in the study.
- The reviewers thought that adding a figure/table reporting the analysis of AEFIs done for area councils and health facilities, and an urban versus rural map and graph with the distribution of facilities and types of AEFIs, respectively, would help identify other relevant patterns in the findings.
- In the reporting of AEFIs, the term “nonserious” may not fully capture the intended meaning. Instead of using this classification, it could be more appropriate to describe the AEFIs reported by mothers/caregivers as “well tolerated” by the infants. This phrasing would avoid the potential ambiguity around what constitutes a “serious” versus “nonserious” AEFI, and instead focus the description on the observed tolerability of the events from the caregiver’s perspective. Adopting this terminology could enhance the clarity and interpretation of the AEFI data presented in the study.
- The description given in the text “Data were received from infants recruited from nine out of the fourteen health facilities, representing primary, secondary, and tertiary institutions across the six Area Councils of Abuja FCT, categorized into urban and rural locations (Table 1 and figure 1 below)” does not appear to be the title of the table. As Figure 1 seems to present the percentage of AEFIs across the three vaccine administration time points, rather than information about the health facility locations. The connection between the text’s description and the actual figure content is unclear and requires clarification.
- It would be helpful if the report provided more information on how the study handled cases where the mother withdrew consent or chose to discontinue their participation.
- The report would benefit from a more detailed caption for Figure 1 that clearly explains the data and visualization being presented.
- The column headers in the tables refer to “recruited babies,” but this terminology may not accurately reflect the study population. It appears these are the infants who completed the full 3-dose vaccination schedule and had their caregivers provide information about AEFIs. To better represent this, the headers could be revised to use more descriptive terms such as “study participants” or “enrolled infants” to highlight that these are the subjects who contributed data to the analysis, rather than simply those who were initially recruited.
- In the Discussion section, it would be helpful to provide references and examples of other countries similar to Nigeria that have already adopted the pentavalent vaccine.
- The study appropriately acknowledges several key limitations; however, there is an opportunity to further strengthen the discussion by exploring the potential influence of additional external factors, such as environmental influences, routine immunization vaccine details, health care access disparities, and socioeconomic conditions, which could have impacted vaccine safety and the reporting of AEFIs. Additionally, incorporating an analysis of the potential influence of maternal health and vaccination history on the infants’ responses to the vaccine could provide a more nuanced understanding of the results, shedding light on how individual-level factors may have contributed to the observed AEFI profiles. By expanding the Limitations section to systematically address these external and individual-level considerations, the study would present a more comprehensive and contextual analysis, enabling readers to better interpret the findings and their broader implications for vaccine safety and AEFI reporting within the target population.

- If similar studies have been conducted elsewhere, a comparison with the findings and protocols used in those studies would make the discussion richer.
- Given the study limitations—particularly due to a lower sample size than expected for a robust analysis—reviewers suggest caution in generalizing the findings.

## Abbreviations

AEFI: adverse event following immunization
